# Influence of different anesthetic and analgesic methods on early cognitive function of elderly patients receiving non-cardiac surgery

**DOI:** 10.12669/pjms.322.9555

**Published:** 2016

**Authors:** Yong Wang, Jie Zhang, Shuijun Zhang

**Affiliations:** 1 Yong Wang, Department of Anesthesiology, The First Affiliated Hospital of Zhengzhou University, Zhengzhou, 450052, China. Key-Disciplines Laboratory Clinical-Medicine Henan, Zhengzhou, 450052, China; 2 Jie Zhang, Department of Anesthesiology, The First Affiliated Hospital of Zhengzhou University, Zhengzhou, 450052, China. Key-Disciplines Laboratory Clinical-Medicine Henan, Zhengzhou, 450052, China; 3 Shuijun Zhang, Department of Hepatopancreatobiliary Surgery, The First Affiliated Hospital of Zhengzhou University, Zhengzhou, 450052, China. Key-Disciplines Laboratory Clinical-Medicine Henan, Zhengzhou, 450052, China

**Keywords:** Anesthesia, Neurological function, Postoperative cognition impairment

## Abstract

**Objective:**

To discuss over influence of two different anesthetic and analgesic methods on early cognitive function of elderly patients who received non-cardiac surgery.

**Methods:**

Two hundred and six elderly patients who underwent non-cardiac surgery were selected as research subjects. They were randomly divided into observation group (103 cases) and control group (103 cases). Patients in observation group were given combined spinal and epidural anesthesia and epidural analgesia, while patients in control group adopted general anesthesia and intravenous analgesia. Neurological function test was carried out one day before surgery and on the 7^th^ day after surgery. Moreover, changes of postoperative pain degree, neuropsychological function and cognitive function were observed and compared.

**Results:**

On the 7^th^ day after surgery, incidence of cognition impairment in observation group and control group was 48.50% (50/103 cases) and 44.70% (46/103 cases), and difference between groups had no statistical significance. Visual Analogue Scale (VAS) Score of observation group was much lower than control group in the 12^th^, 24^th^ and 48^th^ h after surgery (p < 0.05). Logistic regression analysis suggested that, short education years and general surgery were independent risk factors for early cognition impairment.

**Conclusion:**

About 46.60% elderly patients undergoing non-cardiac surgery developed cognition impairment, but influence of different anesthetic and analgesic methods on incidence of postoperative cognition impairment of elderly patients had no significant difference.

## INTRODUCTION

Postoperative cognitive dysfunction (POCD), a commonly seen neurological complication for elderly patients, refers to degradation of memory, orientation, abstract thinking and social activity function after operative anesthesia.^1^,^2^ POCD is mostly temporary; however, some patients develop long-term cognition impairment after surgery, and some patients may even have permanent cognition impairment.^3^,^4^ Occurrence of POCD can delay function recovery, prolong length of stay in hospital, which may bring severe influence on physical recovery and daily life of patients.^5^

Currently, pathogenesis of POCD remains unclear. Usually, POCD is considered as degenerative change of neurological function induced by external causes such as anesthesia and surgery based on degeneration of central nervous system.^6^ It has been reported that,^7^ local anesthesia and general anesthesia have remarkably different influence on physiological function of patients. Therefore, influence of drugs and analgesic method on POCD of elderly patients should be fully considered and evaluated to enable patients to undergo surgery in the best state and avoid POCD effectively, when we select anesthetic drug and analgesic method for elderly patients.^8^,^9^

In this study, we compared the influence of two different anesthetic and analgesic methods on POCD of elderly patients who underwent non-cardiac surgery.

## METHODS

### General data

Two hundred and six elderly patients who received non-cardiac surgery were selected as research objects. They were randomly divided into observation group and control group. Patients over 66 years of age and would undergo non-cardiac surgery below abdomen. In observation group, 65 cases were male and 38 cases were female, with age ranging from 65 years to 78 years (average 71.22 ± 7.87 years) and body mass index (BMI) ranging from 18.40 ~ 25.10 kg/m^2^ (average 23.43 ± 2.54) kg/m^2^ (Classification of Anesthesia Risk: level I ~ II). In control group, 66 were male and 37 were female, with age ranging from 65 years to 80 years (average 71.90 ± 8.44 years) and BMI ranging from 18.73 to 25.12 kg/m^2^ Classification of Anesthesia Risk: level I ~ II). Patients who had heart associated disease, were allergic to anesthetic and analgesic drugs or had allergic constitution, had other surgical contraindication or were unwilling to participate in the research or cooperate were excluded. General data such as gender, age, physical condition and surgical type had no significant difference between two groups (*p* > 0.05); therefore, results were comparable. The study has been approved by the ethical committee of the hospital. Data collected in the study were all used for scientific research and would not be leaked out.

### Method

All patients were forbidden to eat and drink. Patients breathed in oxygen through mask after entering into operation room. After venous channel was opened, blood pressure, electrocardiogram, degree of blood oxygen saturation and end-tidal carbon dioxide were measured. Patients in control group were given general anesthesia and intravenous analgesia; 0.05 mg/kg midazolam, 5 μg/kg fentanyl, 0.30 mg/kg etomidate and 0.10 mg/kg vecuronium bromide were injected as anesthesia induction. Anaesthesia machine was connected after trachea cannula was fulfilled. Anaesthesia was maintained with pump injection of propofol and discontinuous intravenous push of vecuronium bromide and fentanyl. Liquid or blood was supplemented according to hemorrhage during operation. Patients in observation group received combined spinal epidural anesthesia and epidural analgesia. Puncture was performed between the 2nd and 3rd lumbar vertebra. 2 ml bupivacaine (0.50%) was injected into subarachnoid space. 1.50% lidocaine was injected as maintenance anesthesia to prevent obvious pain relief. During operation, changes of vital sings of patients were monitored.

### Observation index

Neuropsychological test:^10^ neuropsychological tests were carried out one day before surgery and on the 7^th^ day after surgery. If neuropsychological function of patients reduced for more than 20%, then neuropsychological function was determined to be degenerated. If two or more deficits were found, then POCD could be confirmed. Pain was evaluated using Visual Analogue Scale (VAS), once every 12 hour, for three times. Intraoperative monitoring: various indexes including medication, amount of bleeding and volume of blood transfusion during operation were recorded.

### Statistical analysis

Data were processed by SPSS 19.0. Comparison of measurement data within group was performed using t test. Comparison of measurement data within group was performed using independent sample t test or Mann-Whitney U test. Enumeration data were compared using chi-square test or Fisher’s exact test. After initial analysis, Logistic regression analysis was made taking whether POCD occurs as dependent variable and factors that may influence occurrence of POCD as independent variable, thus to screen out factors that influence occurrence of POCD. Difference was considered to be significant if *p* < 0.05.

## RESULTS

### Comparisons of intraoperative variables of patients before and during surgery

Duration of anesthesia and duration of operation in observation group were both much longer than control group (*p* < 0.05). That might be associated to different anesthetic methods. Epidural puncture and observation of test dose in observation group required much more time and medication of two groups also had significant difference, but surgical category, amount of bleeding and volume of blood transfusion during operation had no remarkable difference between two groups (Table-I).

**Table-I:** Comparison of the intraoperative variables (Mean ± SD).

Variable	Control group (n=103)	Observation group (n=103)
Duration of anesthesia (min)	193±124	221±108*
Intraoperative anesthetics		
Fentanyl (mg)	/	0.13±0.03
Remifentanil (mg)	1.10±0.60	/
Propofol (mg)	116±64	102±28
Lidocaine (mg)	/	526±310
Morphine (mg)	3.4±0.9	/
Duration of surgery (min)	163±121	192±107*
Intraoperative bleeding (mL)	298±489	334±485
Intraoperative transfusion (mL)	96±290	130±304
Classification of operation		
Operation of general surgery	89(86.40)	78(75.70)
Operation of urology	14(13.60)	25(24.30)

* means p < 0.05 compared to control group.

### VAS score of patients after surgery

VAS score of patients in two groups is shown in Table-II. VAS score of patients in observation group was much lower than control group in the 12^th^, 24^th^ and 36^th^ hour after surgery, and difference was obvious (*p* < 0.05).

**Table-II:** Comparison of VAS score between two groups (Mean ± SD, point).

Group	No.	12h	24h	36h
Observation group	103	1.72±0.88*	1.67±0.78*	1.57±0.69*
Control group	103	2.41±1.19	2.09±0.97	2.01±0.89

* p < 0.05 compared to control group.

### Comparison of neuropsychological function changes between two groups

Through neuropsychological test, we found incidence of POCD was 48.50% in observation group and 44.70% in control group. Neurological deterioration percentage and POCD (2 or more deficits) incidence had no significant differences between two groups (Table III). Incidence of early POCD of all patients was 46.60%.

**Table-III:** Comparison of incidence of neurological deterioration after surgery between two groups [n, (%)]

Degree of deficit	Control group (n=103)	Observation group (n=103)
Patients with 1 deficit	36(35.00)	32(31.00)
Patients with 2 deficits	30(29.10)	32(31.00)
Patients with 3 deficits	10(9.70)	6(5.80)
Patients with 4 deficits	2(2.00)	12(11.70)
Patients with 5 or more deficits	4(3.90)	0(0)
POCD (patients with 2 or more deficits)	46(44.70%)	50(48.50%)

## DISCUSSION

POCD, a commonly seen central nervous system complication for elderly patients, can impact operation result, increase mortality risk and complications, delay recovery, prolong length of stay in hospital and increase expense, which causes adverse influence to patients and also increase burden on society and hospital.^11^,^12^ Currently, an unified conclusion of pathogenesis of POCD has not been drawn by the experts.^13^ It is extensively believed that, POCD is mostly caused by degeneration of central nervous system of patients. During operation, anesthesia can produce external influence on neurological function of patients and thus lead to degenerative changes.^14^ Moreover, surgery will disorder endocrine system, central nervous system and immunologic function, which increases the risk of POCD. A study demonstrates that,^15^ physiological function such as cerebral blood flow, brain metabolism and oxygen delivery is greatly influenced after patients are given general anesthesia. Hence someone guesses that, general anesthesia is more likely to induce POCD in elderly patients than local anesthesia. Research results suggested that, 46.60% elderly patients who underwent non-cardiac surgery developed POCD, but the difference of incidence between groups was insignificant, suggesting influence of different anesthetic and analgesic methods on incidence of POCD had no statistically significant difference.

Correlation between anesthetic method and POCD has been disputed for years.^16^ A prospective random study of Williams-Russo et al. once compared influence of epidural anesthesia and general anesthesia on incidence of POCD in elderly patients and found incidence of POCD in patients receiving different anesthetic methods had no remarkable difference in short term (one week) and long term (six months).^17^ Rasmussen et al. found that,^18^ patients who had local anesthesia were much less likely to have POCD in early stage after surgery (one week later) compared to general anesthesia, but the incidence of POCD in three months after surgery between two groups had no remarkable different. Wu et al. once retrospectively analyzed relevant literature and found intraspinal anesthesia could not lead to lower incidence of POCD.^19^ Postoperative pain is also a risk factor of POCD. But few researches involve influence of postoperative analgesia on POCD.^20^,^21^ This study compared influence of general anesthesia in combination with intravenous analgesia and combined general and epidural anesthesia in combination with postoperative epidural analgesia on incidence of early POCD of patients undergoing non-cardiac surgery. Combined general and epidural anesthesia is extensively applied in recent years; and epidural analgesia can relieve stress reaction stimulated by operation and provide more effectively eliminate pain of patients, which is more beneficial for reducing incidence of early POCD after surgery theoretically. The current study also suggested that, VAS score of observation group was much lower than control group, indicating general anesthesia in combination with intravenous analgesia was less effective than combined spinal and epidural anesthesia and epidural analgesia. But influence of two methods on incidence of postoperative complications and POCD had no remarkable difference.

## CONCLUSION

To sum up, elderly patients who undergo non-cardiac surgery have a high probability to develop POCD, but influence of different anesthetic and analgesic methods on POCD had no obvious difference. Combined spinal and epidural anesthesia and epidural analgesia can effectively lower VAS score, and patients who adopt that anesthetic and analgesic method have a similar incidence with general anesthesia; therefore, it deserves more application.
